# Two Key Residues in EphrinB3 Are Critical for Its Use as an Alternative Receptor for Nipah Virus

**DOI:** 10.1371/journal.ppat.0020007

**Published:** 2006-02-10

**Authors:** Oscar A Negrete, Mike C Wolf, Hector C Aguilar, Sven Enterlein, Wei Wang, Elke Mühlberger, Stephen V Su, Andrea Bertolotti-Ciarlet, Ramon Flick, Benhur Lee

**Affiliations:** 1 Department of Microbiology, Immunology and Molecular Genetics, David Geffen School of Medicine, University of California Los Angeles, Los Angeles, California, United States of America; 2 University of Texas Medical Branch, Galveston, Texas, United States of America; 3 Department of Medicine-Infectious Diseases, David Geffen School of Medicine, University of California Los Angeles, Los Angeles, California, United States of America; 4 Institute for Virology, Philipps-University Marburg, Marburg, Germany; 5 Department of Microbiology, University of Pennsylvania, Philadelphia, Pennsylvania, United States of America; 6 Department of Pathology and Laboratory Medicine, David Geffen School of Medicine, University of California Los Angeles, Los Angeles, California, United States of America; 7 UCLA AIDS Institute, David Geffen School of Medicine, University of California Los Angeles, Los Angeles, California, United States of America; Robarts Research Institute, Canada

## Abstract

EphrinB2 was recently discovered as a functional receptor for Nipah virus (NiV), a lethal emerging paramyxovirus. Ephrins constitute a class of homologous ligands for the Eph class of receptor tyrosine kinases and exhibit overlapping expression patterns. Thus, we examined whether other ephrins might serve as alternative receptors for NiV. Here, we show that of all known ephrins (ephrinA1–A5 and ephrinB1–B3), only the soluble Fc-fusion proteins of ephrinB3, in addition to ephrinB2, bound to soluble NiV attachment protein G (NiV-G). Soluble NiV-G bound to cell surface ephrinB3 and B2 with subnanomolar affinities (K_d_ = 0.58 nM and 0.06 nM for ephrinB3 and B2, respectively). Surface plasmon resonance analysis indicated that the relatively lower affinity of NiV-G for ephrinB3 was largely due to a faster off-rate (K_off_ = 1.94 × 10^−3^ s^−1^ versus 1.06 × 10^−4^ s^−1^ for ephrinB3 and B2, respectively). EphrinB3 was sufficient to allow for viral entry of both pseudotype and live NiV. Soluble ephrinB2 and B3 were able to compete for NiV-envelope-mediated viral entry on both ephrinB2- and B3-expressing cells, suggesting that NiV-G interacts with both ephrinB2 and B3 via an overlapping site. Mutational analysis indicated that the Leu–Trp residues in the solvent exposed G–H loop of ephrinB2 and B3 were critical determinants of NiV binding and entry. Indeed, replacement of the Tyr–Met residues in the homologous positions in ephrinB1 with Leu–Trp conferred NiV receptor activity to ephrinB1. Thus, ephrinB3 is a bona fide alternate receptor for NiV entry, and two residues in the G–H loop of the ephrin B-class ligands are critical determinants of NiV receptor activity.

## Synopsis

Nipah virus is a deadly virus that can cause death in up to 70% of infected patients, mostly from fatal inflammation of the brain. Nipah virus is considered a “priority pathogen” for bioterrorism purposes, and it has the potential for widespread economic devastation as it can spread rapidly among susceptible livestock. The authors had previously identified the receptor that mediates Nipah virus entry into cells. This receptor, ephrinB2, is a critical molecule for the development of the vascular and nervous system and is highly expressed on endothelial cells and neurons, which are also the two cell types preferentially infected by Nipah virus in vivo.

EphrinB2 belongs to a large family of related molecules that are variably conserved in structure and function. Thus, the authors screened all known ephrins, and found that a closely related molecule, ephrinB3, also can function as an entry receptor for Nipah virus. In addition, the authors established that while ephrinB2 was better used than ephrinB3 as an entry receptor, the same two critical amino acids in ephrinB2 and B3 were responsible for the viral receptor activity of these molecules. The discovery of a more comprehensive set of NiV receptors will aid our understanding of the pathology underlying NiV disease.

## Introduction

Nipah virus (NiV) is a zoonotic paramyxovirus classified in the taxonomic unit *Henipavirus* under the family of *Paramyxoviridae* [1,2]. NiV emerged in peninsular Malaysia and Singapore in 1998–1999 when cases of severe acute encephalitis occurred among agricultural and abattoir workers in close contact with NiV-infected pigs [3]. In 2004 two confirmed outbreaks of NiV in Bangladesh recorded mortality rates of greater than 70% with evidence of human-to-human transmission [4,5]. Due to high mortality rates and its potential use as a bioweapon [6], NiV has been classified as a Category C priority pathogen for biodefense purposes. NiV not only has the potential to cause severe disease in humans, but also represents a critical economic threat if used against the pig-farming industry.

Pathological investigations of NiV-infected patients revealed that a major cellular target of the NiV appears to be endothelial cells that line blood vessels [7]. More important, syncytial or multinucleated giant endothelial cells were seen in the microvasculature of many organs, with the most severe damage occurring to vessels in the central nervous system (CNS). Concordantly, NiV antigen load was highest in the brain parenchyma, especially in neurons, compared with other organs. The functional receptor for NiV entry, ephrinB2 [8,9], is expressed on endothelial cells and neurons [10,11] consistent with the known cellular tropism for NiV [7,12].

Ephrins are the highly conserved ligands to the Eph family of receptor tyrosine kinases [13]. Eph–ephrin signaling functions in both embryonic and adult tissues by regulating processes such as angiogenesis, neuron axonal guidance, and tumorgenesis [11,13–15]. Both ephrins and Eph receptors are categorized into class-A and class-B proteins based on sequence homology, binding affinities, and the manner of ephrin membrane attachment. In general, ephrin ligands and receptors can interact promiscuously inside their own class but not outside of it. An exception to the rule is the binding of EphA4 to ephrinB3, an interaction thought to be important in corticospinal neurons crossing the spinal cord midline [16,17]. Ephrins have extremely conserved features, especially within a class, and can have overlapping but distinct expression patterns [18].

Here, we examined whether the NiV attachment protein G (NiV-G) can act as promiscuously as the Eph receptors in binding to other ephrins, in addition to ephrinB2. We showed that ephrinB2 and B3 bound to NiV-G with subnanomolar affinities. Expression of human ephrinB3 in nonpermissive CHO-pgsA745 cells rendered them permissive to NiV entry and infection. Lastly, we characterized the important conserved features of ephrinB2 and B3 that account for their NiV receptor activity. The discovery of a more comprehensive set of NiV receptors will aid our understanding of the pathology underlying NiV disease.

## Results

### NiV-G Binds EphrinB3 at Lower Affinity than EphrinB2

**Figure 1:**
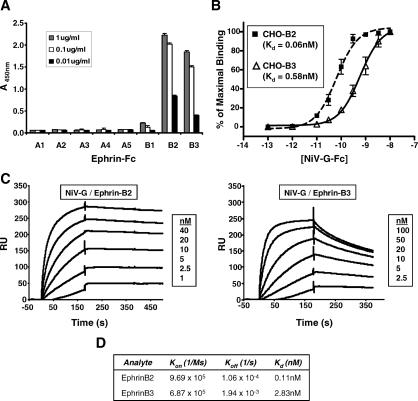
Soluble NiV-G Binds to EphrinB3 with Lower Affinity than EphrinB2 (A) 1.0 μg/ml, 0.1 μg/ml, and 0.01 μg/ml of the indicated ephrin-Fc fusion proteins were allowed to bind to soluble NiV-G-coated plates in an ELISA format (see Materials and Methods). The amount of ligand bound was detected colorimetrically using an antihuman Fc antibody conjugated to horseradish peroxidase. One representative experiment out of three is shown. Data are averages of triplicates ± standard error (SE). (B) EphrinB2 and B3 stably transfected CHO-pgsA745 cells (CHO-B2 and CHO-B3, respectively) were used to measure NiV-G-Fc cell surface binding. Increasing concentrations of NiV-G-Fc were added to either CHO-B2 cells (dashed line with squares) or CHO-B3 cells (solid line with triangles), and binding was assessed by flow cytometry using R-phycoerythrin-conjugated anti-Fc antibodies. Regression curves were generated as described in Materials and Methods. Each data point is an average ± SE from three experiments. (C) Surface plasmon resonance (BIAcore 3000) measured the binding kinetics of NiV-G-Fc to both ephrinB2-Fc and ephrinB3-Fc in response units (RU). NiV-G-Fc was immobilized to a CM5 sensor chip via an amide coupling procedure, and increasing concentrations of ephrinB2-Fc and ephrinB3-Fc were flowed as analyte over the sensor chip. One representative experiment out of two is shown. (D) K _d_, K _on_ (association-rate), and K _off_ (dissociation-rate) were determined by fitting the binding chromatogram data from (C) with BIAcore evaluation software (version 3.1) using the 1:1 Langmuir binding model.

Ephrins constitute a highly conserved class of proteins with many homologous members. Thus, we examined if any ephrins, other than ephrinB2, can bind similarly to NiV-G. Using an enzyme-linked immunosorbent assay (ELISA), we screened for the ability of soluble HA-tagged ectodomain of NiV-G (NiV-G-HA) to bind to all known ephrins (ephrinA1–A5 and ephrinB1–B3). We found that ephrinB3-Fc, in addition to ephrinB2-Fc, bound to NiV-G-HA (Figure 1A). To confirm these results with cell surface-expressed ephrins, CHO-pgsA745 cells were stably transfected with human ephrinB1 (CHO-B1), ephrinB2 (CHO-B2), or ephrinB3 (CHO-B3). Chinese hamster ovary (CHO) cells do not express ephrins endogenously [19], while the CHO-pgsA745 cells derived from these CHO cells lack heparin sulfate proteoglycans [20]. Heparin sulfates are known to act as entry or attachment receptors for many viruses, which can confound viral receptor studies. Thus, we used NiV-G-Fc, a fusion construct between the ectodomain of NiV-G and the Fc region of human IgG1, to measure the binding of NiV-G to each of the ephrin B-class ligands stably expressed on CHO-pgsA745 cells (Figure 1B). Remarkably, the K_d_ for NiV-G-Fc binding to ephrinB2 and B3 was in the subnanomolar range (K_d_ = 0.06 nM and 0.58 nM for ephrinB2 and B3, respectively). NiV-G-Fc did not bind to CHO-B1 cells when tested under the same conditions used for CHO-B2 and CHO-B3 cells (unpublished data).

To delineate the difference in binding affinities between ephrinB2 and B3, we examined their binding kinetics to NiV-G-Fc. We performed surface plasmon resonance analysis by coupling NiV-G-Fc to the sensor chip (as ligand) while using soluble ephrinB2 and B3 as analyte. BIAcore analysis of NiV-G-Fc binding to ephrinB2-Fc and B3-Fc indicated that while NiV-G-Fc bound to ephrinB2 and B3 with similar on-rates (K_on_ = 9.69 × 10^5^ and 6.87 × 10^5^ for ephrinB2 and B3, respectively), their off-rates were significantly different (Figure 1C and 1D). The K_off_ for ephrinB2-Fc binding to NiV-G-Fc (1.06 × 10^−4^ s^−1^) was more than 10-fold slower than ephrinB3-Fc (1.94 × 10^−3^ s^−1^) binding (Figure 1D). EphrinB1-Fc was also tested similarly to ephrinB2 and B3 and did not exhibit any binding to NiV-G-Fc even up to concentrations as high as 500 nM (unpublished data). As a control to determine the accuracy of our BIAcore measurements, we determined the K_d_ of EphB4-Fc binding to ephrinB2-Fc to be 0.37 nM, a value consistent with published values of approximately 0.5 nM [21] (unpublished data). Cumulatively, our data show that the Nipah attachment protein bound to both ephrinB2 and ephrinB3 with different but significant affinities.

### EphrinB3 Supports NiV Entry and Infection

**Figure 2:**
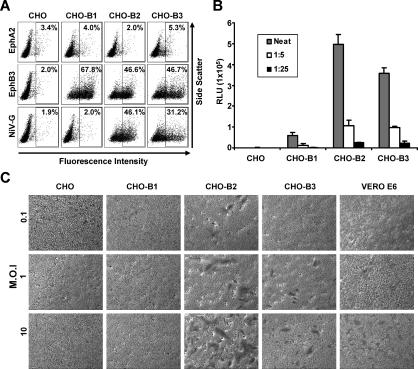
Pseudotyped and Live NiV Use EphrinB2 and B3 for Cellular Entry (A) Ephrin expression was measured by flow cytometry on CHO-pgsA745 parental cells (CHO) and CHO-pgsA745 cells stably expressing ephrinB1, B2, and B3 (CHO-B1, CHO-B2, and CHO-B3). To bind the CHO cell lines, 10 μg/ml of EphA2, 10 μg/ml EphB3-Fc, and 1 nM of NiV-G-Fc were used, and the amount of binding was detected by flow cytometry as in Figure 1B. Data are representative of three experiments. (B) NiV-F and G glycoproteins were pseudotyped onto a VSV-ΔG-Luc core virus (NiV-VSV-ΔG-Luc) and used to infect parental CHO-pgsA745 (CHO), CHO-B1, CHO-B2, and CHO-B3 cells. Entry of the indicated dilutions of NiV-VSV-ΔG-Luc viruses was measured by quantifying *Renilla* Luc activity according to manufacturer's directions. Relative light units (RLU) were acquired and quantified on a Veritas luminometer. Data are shown as averages of triplicates ± standard deviation of a representative experiment. In three independent experiments, viral entry into CHO-B3 cells was reduced by 21%, 28%, and 46%, respectively, compared to CHO-B2 cells (*p* = 0.05, paired *t*-test). (C) The listed MOIs of live NiV were used to infect the indicated cell lines (10^5^ cells per infection). Foci of syncytia were observed 24 h postinfection. Vero E6 cells are fully permissive for NiV infection and were used as positive control cells. Note the larger number of syncytia seen on CHO-B2 versus CHO-B3 cells.

We next examined whether the high-affinity protein interaction seen between ephrinB3 and NiV-G was sufficient to permit the entry of NiV. We first established a panel of CHO-pgsA745 cells stably expressing ephrinB1, B2, and B3. Since the EphB3 receptor binds to all ephrin B-class ligands with similar affinities (0.27–1.8 nM for ephrinB1, 0.28–0.78 nM for ephrinB2, and 1.5 nM for ephrinB3) [21], we used saturating amounts of soluble EphB3 (EphB3-Fc) to determine the level of ephrinB1–B3 expression in our stable cell lines by flow cytometry (Figure 2A). We found that while CHO-B1, CHO-B2, and CHO-B3 cells were significantly positive for EphB3 binding (68%, 47%, and 47%, respectively), only CHO-B2 and CHO-B3 cells bound to NiV-G-Fc (46% and 31%, respectively). Soluble EphB3 did not bind to parental CHO-pgsA745 cells, nor did the ephrin A-class-specific EphA2-Fc bind any of the cell lines tested (CHO-pgsA745, CHO-B1, CHO-B2, or CHO-B3). These results confirm the specific ephrin B-class expression on our panel of CHO-pgsA745 cells.

We then proceeded to quantitate NiV entry using NiV envelope pseudotyped luciferase (Luc) reporter viruses. We had previously shown that NiV envelopes can be successfully pseudotyped onto recombinant vesicular stomatitis virus (VSV) expressing a red fluorescent protein (RFP), but lacking its own envelope (NiV-VSV-ΔG-RFP) [8]. Here, the NiV-VSV-ΔG-Luc was made bearing the NiV fusion (F) and NiV-G and expressing the *Renilla* Luc reporter gene in place of the RFP gene. These NiV envelope pseudotyped VSV particles were used to infect CHO-pgsA745 parental cells (CHO), CHO-B1, CHO-B2, and CHO-B3 cell lines. We found that both CHO-B2 and CHO-B3 allowed entry of NiV-VSV-ΔG-Luc virus (Figure 2B), although viral entry into CHO-B2 cells reproducibly resulted in higher Luc levels (*p* = 0.05, paired *t*-test). In three independent experiments, the reduction in viral entry into CHO-B3 cells ranged from 21% to 46%. Since the EphB3-Fc binding data indicated similar levels of ephrinB2 and B3 expression on the CHO-B2 and CHO-B3 cells (Figure 2A), NiV-VSV-ΔG-Luc virus entered CHO-B2 cells more efficiently than CHO-B3 cells.

To examine whether ephrinB3 can support viral infection, live NiV infections were performed under Biosafety Level-4 conditions. The hallmark of NiV infection in humans is the presence of syncytial or multinucleated giant endothelial cells, and cell lines from many different species produced syncytia upon infection. Therefore, we looked for syncytia formation in CHO, CHO-B1, CHO-B2, CHO-B3, and Vero cells after infection with live NiV. Indeed, we found that live NiV can infect ephrinB2- and B3-expressing cells, although ephrinB2 appears to be used more efficiently (Figure 2C). With any given multiplicity of infection (MOI), at 24 h postinfection, there were always a greater number of syncytia in the CHO-B2 versus CHO-B3 cells. No syncytia were detected in CHO-B1 cells. At 48 h postinfection, syncytia were apparent at all MOIs tested in the CHO-B3 cells (unpublished data). Thus, ephrinB3 can serve as a bona fide alternative receptor for NiV entry.

### NiV-G Binds EphrinB2 and B3 via an Overlapping Site

**Figure 3:**
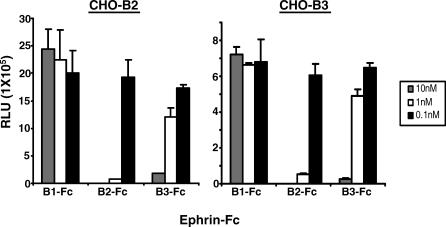
EphrinB2 and B3 Bind NiV-G at an Overlapping Site NiV-VSV-ΔG-Luc pseudotyped viruses were used to infect CHO-B2 and CHO-B3 cells in the presence of the indicated amounts of ephrinB1, B2, and B3-Fc fusion proteins (B1-Fc, B2-Fc, and B3-Fc, respectively). Entry was measured as in Figure 2A. Data are the average of triplicates ± standard deviation, and one representative experiment of three is shown.

Next, we asked whether ephrinB2 and B3 interact with NiV-G in a distinct or overlapping manner. To answer this question, we used a competition assay where CHO-B2 and CHO-B3 cells were infected with NiV-VSV-ΔG-Luc viruses in the presence of soluble ephrin B-class ligands. As expected, ephrinB2-Fc inhibited pseudotyped NiV on CHO-B2 cells while ephrinB1-Fc did not inhibit entry (Figure 3, left). However, ephrinB3-Fc also inhibited pseudotyped NiV entry on CHO-B2 cells, suggesting that ephrinB3 blocked ephrinB2-dependent NiV entry by competing for a similar binding domain on NiV-G. Conversely, ephrinB2-Fc also inhibited pseudotyped NiV entry on CHO-B3 cells (Figure 3, right). In both CHO-B2 and CHO-B3 cells, ephrinB2-Fc was a more effective inhibitor of NiV-G entry than ephrinB3-Fc. In summary, these results suggest that ephrinB2 and B3 binding sites on NiV-G are overlapping.

### Leu–Trp Residues in the G–H Loop of EphrinB2 and B3 Are Critical for NiV-G Binding

**Figure 4:**
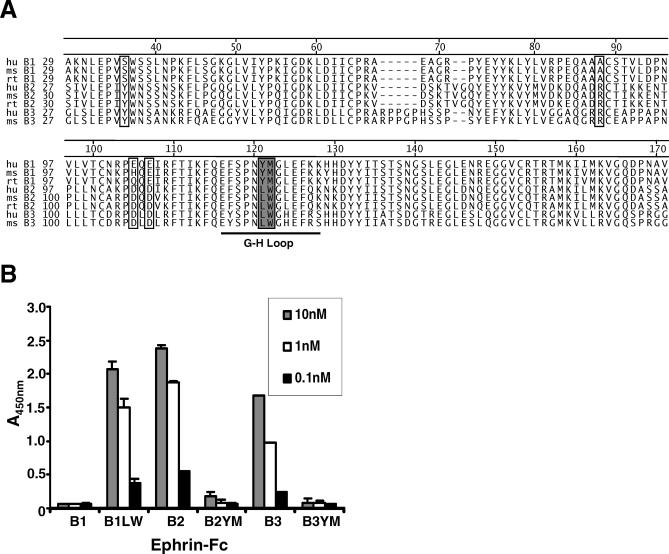
The Leu–Trp Residues Present in the G–H Loop of EphrinB2 and B3 Are the Critical Determinants of NiV-G Binding (A) Sequence alignment of human (hu), mouse (ms), and rat (rt) ephrinB1, B2, and B3 ectodomains using the Jotun Hein algorithm (DNAstar Megalign software). Six residues in the ephrin B-class ectodomain reveal solvent-exposed amino acids [22] that contain identical residues in both ephrinB2 and B3 but different residues in ephrinB1 (open box). Examination of the ephrin binding loop (G–H loop) indicates the L–W residues in ephrin B2 and B3 are replaced by Y–M residues in ephrinB1 (filled box). (B) Ephrin-Fc mutants were created by substituting the L–W residues present in ephrinB2 and B3 with Y–M residues using site-directed mutagenesis (B2YM-Fc and B3YM-Fc). Conversely, the Y–M residues in ephrinB1 were exchanged for the L–W residues (B1LW-Fc); 10 nM, 1 nM, and 0.1 nM of both wild-type (B1, B2, and B3) and mutant (B1LW, B2YM, and B3YM) ephrin-Fc proteins were tested for their ability to bind NiV-G-HA in an ELISA. The amount of binding was measured the same as in Figure 1A. The data are averages of three experiments done in triplicates ± standard error.

Since ephrinB2 and B3 can support NiV entry, while ephrinB1 cannot, we hypothesize that conserved residues common in both ephrinB2 and B3 mediate specific interactions with NiV-G. Alignment of human, mouse, and rat ephrinB1, B2, and B3 sequences identified common residues in ephrinB2 and B3 not present in ephrinB1 (Figure 4A). Examination of the homologous G–H loop regions between ephrinB1, B2, and B3 reveals that the L–W (Leu–Trp) residues present in ephrinB2 and B3 are replaced by Y–M (Tyr–Met) in ephrinB1 (Figure 4A). The cocrystal structure of ephrinB2 and the EphB2 receptor (an endogenous ephrinB2 receptor) indicates that the L–W (Leu_124_–Trp_125_) residues in the G–H loop of ephrinB2 insert deep into a hydrophobic pocket in EphB2 [22]. We believe this interaction to be informative as soluble EphB2 inhibits NiV-G mediated infection[8] and is likely to interact with a similar region on ephrinB2 as NiV-G. Thus, we sought to determine if the G–H loop L–W residues in ephrinB2 and ephrinB3 also interact with NiV-G.

Full-length human ephrinB1, B2, and B3 clones were obtained, and the ectodomain of each was fused to the Fc region of human IgG1. Replacement of Y–M in ephrinB1 with L–W from the homologous positions in ephrinB2 and B3 resulted in a soluble ephrinB1 mutant, B1_LW_-Fc, that bound NiV-G almost as well as ephrinB2-Fc (Figure 4B). Conversely, the L–W to Y–M mutations in soluble ephrinB2 (B2_YM_-Fc) and ephrinB3 (B3_YM_-Fc) abrogated binding to NiV-G-HA, although at higher concentrations, B2_YM_-Fc appears to retain minimal NiV-G binding activity.

### Leu–Trp Residues in the G–H Loop of EphrinB3 Are Necessary for NiV Entry

**Figure 5:**
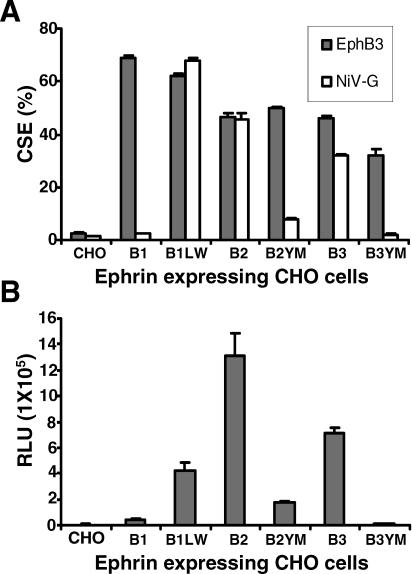
The Leu–Trp Residues in G–H loop of EphrinB3 Are Necessary for Pseudotyped NiV Entry (A) The percentage of ephrin cell surface expression (CSE) was measured by flow cytometry on CHO-pgs745 parental cells (CHO) and CHO-pgs745 cells stably expressing both full-length wild-type ephrins (B1, B2, and B3) and mutant ephrins (B1LW, B2YM, and B3YM); 10 μg/ml of EphB3-Fc (solid bar) and 1 nM of NiV-G-Fc (open bar) were used to bind the CHO cell lines, and the amount of binding was detected the same as in Figure 1B. The data are an average of triplicates ± standard deviation (SD). (B) The same CHO cell lines used above were seeded at 10^5^ cells per well and infected with pseudotyped NiV-VSV-ΔG-Luc virus. The amount of entry was detected as in Figure 2A. One representative experiment of three is shown, and data are an average of triplicates ± SD. In three independent experiments, the viral entry into B2YM cells was reduced by 45%, 68%, and 85%, respectively, compared to wild-type B2 cells (*p* < 0.03, paired *t*-test).

Next, we examined the effects of these L–W/Y–M mutations in the context of full-length ephrins and their ability to support NiV entry. To do so, we first made stable CHO-pgsA745 cells expressing the full-length ephrin mutants (CHO-B1_LW_, CHO-B2_YM_, and CHO-B3_YM_). We compared the level of mutant ephrin expression in the stable cell lines to that of wild-type ephrin expression using EphB3-Fc in a flow cytometry analysis. It is unlikely that EphB3 binds to the ephrin LW/YM residues in question since EphB3 can bind ephrinB1, B2, and B3 with similar affinities [21]. Therefore, using EphB3-Fc to measure cell surface expression, we found that both wild-type ephrin and its relevant mutant were expressed at similar levels (Figure 5A; compare B1 to B1_LW_, B2 to B2_YM_, and B3 to B3_YM_). Parental CHO-pgsA745 (CHO) served as a negative control and soluble EphB3 did not bind to these cells. In addition, NiV-G-Fc bound to the mutant ephrins in the expected patterns seen in our previous solid state ELISA experiment (Figure 4B).

We then proceeded to infect these cells with NiV-VSV-Luc pseudotyped viruses (Figure 5B). As expected, both ephrinB2 and B3 permitted NiV envelope-mediated entry as described previously (Figure 2B). The B1_LW_ mutant now supported NiV entry, while entry into B2_YM_ cells was markedly reduced. Entry into B3_YM_ was completely abrogated. We note that while entry into B2_YM_ cells was reduced by about 85% compared to wild-type ephrinB2 cells, entry was still 44-fold over background (parental CHO-pgsA745 cells). These results suggest that while the L_124_–W_125_ residues in ephrinB3 appear to be critical for NiV entry, other residues in ephrinB2 likely have a supporting role in mediating NiV entry. Interestingly, the B1_LW_ mutant could not fully restore entry equivalent to wild-type B2 or B3 levels, even though NiV-G bound to cell surface ephrinB1_LW_ at wild-type ephrinB2 levels. Therefore, in addition to simple binding, additional residues in ephrinB2 likely mediate the subsequent conformational changes in NiV-G and/or F that leads to membrane fusion and entry.

## Discussion

EphrinB2 was recently identified as a functional cellular receptor for NiV [8,9]. EphrinB2 expression on endothelial cells, neurons and smooth muscle cells [10,11] is highly consistent with the known tropism of NiV infection [7]. Here, we show that ephrinB3 is an alternate receptor for NiV and is independently able to support NiV entry and infection, albeit less efficiently than ephrinB2. NiV-G binds to both ephrinB2 and B3 with subnanomolar affinity, with the relatively weaker K_d_ of NiV-G for ephrinB3 explained by its faster off-rate. Finally, we implicate two residues (L–W) common in the G–H loop of ephrinB2 and B3 as crucial for NiV receptor activity. Remarkably, replacement of the Y–M residues in the homologous positions in ephrinB1 with L–W conferred wild-type NiV-G binding activity and substantial NiV receptor activity to a protein that is otherwise nonfunctional as a NiV receptor.

To our knowledge, there is no specific indication that ephrinB3 is expressed in the endothelium. At the minimum, ephrinB3 does not appear to be critical to vascular development since ephrinB3 knockout mice lack the overt defects in vascular morphogenesis seen in ephrinB2 knockout mice [23,24]. However, NiV entry into microvascular endothelial cells is almost completely abrogated by soluble ephB4-Fc [8], which binds to ephrinB2 but not B3, suggesting that ephrinB3 is likely not expressed on endothelial cells, at least not at levels that can support robust viral entry. In contrast, ephrinB3 is expressed in the CNS in overlapping and distinct patterns with ephrinB2 [18,25]. In the regions of the adult brain such as the cerebral cortex [26,27] and the hippocampus [28] where ephrinB2 and B3 exhibit overlapping expression, NiV could potentially use either receptor for entry with a possible preference for ephrinB2 based on the higher affinity of NiV-G for ephrinB2. However, in regions such as the corpus callosum [27] and the spinal cord [16,17], ephrinB3 is distinctly expressed and could account for specific aspects of NiV pathology.

EphrinB3 knockout mice studies indicate ephrinB3 is expressed in the spinal cord midline and functions to prevent corticospinal tract axons from recrossing the midline. Coincidently, in a histological study of NiV infection by Wong et al., three of eight patients examined showed pathological lesions in the spinal cord similar to other regions of the CNS [7]. Indeed, clinical symptoms of segmental myoclonus and flaccid tetraplegia combined with nerve conduction studies have also suggested upper cervical and lower spinal cord involvement [29], and magnetic resonance imaging confirmation of a spinal cord lesion at the cognate C7 level in a patient who developed left-arm dysaesthesia and finger weakness has also been reported [30]. In another study that reported magnetic resonance imaging findings in eight patients with NiV encephalitis, half the patients had numerous punctate lesions in the corpus callosum [31], where ephrinB3, but not ephrinB2, is expressed [27]. Thus, although ephrinB2 seems to be the primary receptor for NiV, ephrinB3 can likely be used as an alternative receptor and may account for some of the CNS pathology seen in NiV infection.

In this study, we also established that NiV-G binding to ephrinB2 and B3 is dependent on the same L–W residues that are important for endogenous ephrinB2/EphB2 interactions [21,22]. Since NiV-G interacts with ephrinB2 in a similar fashion with at least some of the Eph B-class receptors, and NiV-G forms higher order oligomers [32] analogous to Eph B-class receptors [10,11], NiV-G could potentially induce “reverse-signaling” upon ephrinB2 or B3 binding.

In vivo, Eph–ephrin interactions cause bidirectional signaling that can direct the migration of endothelial cells and neuronal dendrites [10,11,33–35]. Therefore, NiV infection may not only target ephrinB2- or B3-expressing cells, but also disrupt normal Eph–ephrin signaling and possibly alter cellular migration patterns. Indeed, infusion of soluble EphB2-Fc has been reported to disrupt the migration of ephrinB2- and B3-expressing cells of the subventricular zone region in an adult mouse [36]. Although the levels of Ephs and ephrins in other regions of the adult brain are reduced compared to neonatal-stage expression [27], Eph and ephrins can alter their expression patterns after injury to the spinal cord [37], hippocampus [38], or after infection [39,40]. In this case, NiV infection may alter ephrin expression patterns in the CNS and disrupt the endogenous Eph–ephrin signaling resulting in the neuropsychiatric [41] or neuropathologic sequelae seen in NiV infections.

Zoonotic diseases such as those caused by NiV have become an increasing threat in several parts of the world [42]. The habitat of the pteropid fruit bat, considered as the natural reservoir host, spans from the east coast of Africa across southern and Southeast Asia, east to the Philippines and Pacific islands, and south to Australia [43]. Although NiV outbreaks have only occurred in Malaysia, Bangladesh, and Singapore, increased surveillance in other geographical regions of the pteropid habitat found bats to harbor NiV [44]. Therefore, NiV continues to remain a potential threat to both human and animal populations. This underscores the need for the development of antiviral therapeutics. A complete understanding of Nipah viral entry at the level of receptor engagement may help in these efforts.

## Materials and Methods

### Cells and culture conditions.

CHO-pgsA745 is a mutant cell line derived from CHO cells that lack the endogenous expression of heparin sulfate proteogylcans [19]. CHO-pgsA745 cells and Vero (African green monkey kidney fibroblasts) cells were maintained in DMEM/F12 and α-MEM (Invitrogen, Carlsbad, California, United States), respectively, and both were supplemented with 10% fetal bovine serum (Omega Scientific, Tarzana, California, United States) and the antibiotics penicillin and streptomycin. CHO-pgsA745 cells expressing either wild-type or mutant ephrins were made by selecting for neomycin resistance with 0.5 mg/ml of G418 after transfection. Once selected, the ephrin-expressing populations were enriched using the magnetic bead selection (Miltenyi Biotech, Auburn, California, United States). Briefly, EphB3-Fc (R & D Systems, Minneapolis, Minnesota, United States) was coupled to protein G microbeads (Miltenyi Biotech), and then 2 × 10^6^ ephrin-expressing CHO-pgsA745 cells were added. Then, the cell–bead mixture was poured over a MACS MS column (Miltenyi Biotech), followed by positive cells elution.

### Plasmids and reagents.

Soluble Fc-fusion ephrin proteins (ephrinA1–A5 and ephrinB1–B3) and Eph proteins (EphA2-Fc and EphB3-Fc) were purchased from R & D Systems. Human ephrinB2 and ephrinB3 plasmids were purchased from GeneCopoeia (Germantown, Maryland, United States), and human ephrinB1 was obtained from Open Biosystems (Huntsville, Alabama, Unites States). Each ephrin open reading frame was subcloned into the pcDNA3.1 vector (Invitrogen) under CMV promoter-driven expression. In-house ephrin-Fc fusion constructs were made by subcloning the ectodomain of each ephrin into the pCR3-Fc vector, which contains the CH2 and CH3 domains of human IgG1. Mutations in both the full-length (pcDNA3.1 clones) and soluble (pCR3-Fc clones) [8,45] were made using the QuikChange (Stratagene, La Jolla, California, United States) site-directed mutagenesis kit. All subclones and mutations were confirmed by sequencing.

### Binding of soluble ephrins to NiV-G.

Supernatant from NiV-G-HA-transfected 293T cells was used to coat MaxiSorp high protein-binding 96-well plates (Nalg Nunc International, Rochester, New York, United States) overnight at 4 °C. The NiV-G-HA-coated plates were then blocked with 5% bovine serum albumin (BSA) in Tris-buffer saline (TBS) for 2 h at 37 °C. The plates were rinsed with wash buffer (1% BSA, 0.05% Tween-20 in TBS), and ephrin-Fc proteins, diluted in wash buffer, were placed in each well to bind soluble NiV-G for 1 h at room temperature. The plates were washed three times with wash buffer and incubated with antihuman Fc monoclonal antibody conjugated with HRP for 30 min at room temperature. The plates were then washed three more times, and the amount of bound ephrin was assessed with 1-step Ultra TMB substrate (Pierce, Rockford, Illinois, United States). The colorimetric reading was performed on a spectrophotometer (Dynex Technologies, Chantilly, Virginia, United States). For each soluble ephrin-Fc, each experiment was performed three times, each time in triplicates.

### Cell surface binding assays.

The ephrinB2- and B3-expressing CHO-pgsA745 cells (CHO-B2 and CHO-B3, respectively) were made as described above. Increasing amounts of the NiV-G-Fc were incubated with CHO-B2 or CHO-B3 cells for 1 h on ice. Then, the cells were washed with buffer and incubated with R-phycoerythrin-conjugated anti-Fc antibodies for 30 min on ice. The cells were washed again and fixed with 2% paraformaldehyde, and the data were collected using a FACScan flow cytometer (Becton Dickinson, Franklin Lakes, New Jersey, United States). The data were analyzed using FCS Express V2 (DeNovo Software, Thornhill, Ontario, Canada). The cell surface K_d_ values were calculated using the GraphPad Prism software (San Diego, California, United States) by normalizing the highest mean fluorescent intensity value obtained to 100%.

### Pseudotyped virus infection assay.

NiV pseudotyped particles were made from the VSV-ΔG-Luc virus, a recombinant VSV derived from a full-length complementary DNA clone of the VSV Indiana serotype in which the G-protein envelope has been replaced with *Renilla* Luc (kindly provided by Andrea Bertolotti-Ciarlet). NiV envelopes F and G were provided in trans, and the VSV-ΔG-NiV pseudotyped viruses were then used to infect the various CHO-pgsA745 cell lines. Briefly, the cells were seeded overnight in a 48-well plate and then infected for 1 h at 37 °C with VSV-ΔG-NiV diluted in 1% FBS in phosphate buffer saline (PBS). The inoculum was removed and washed with PBS and replaced by culture media. The next day, the cells were lysed, the lysates were mixed with *Renilla* Luc subtrate (Promega, Madison, Wisconsin, United States), and a luminescence reading was performed on a Veritas luminometer (Turner BioSystems, Sunnyvale, California, United States). Each condition was done in triplicate and infections were repeated three times.

### Live NiV infection assay.

Parental CHO-pgsA745, ephrin-expressing CHO-pgsA745, and Vero cells were seeded at 10^5^ cells per well in 24-well plates for live NiV infections. In the L4 facility of the Philipps-University Marburg under Biosafety Level 4 (BSL-4) conditions, the cells were then infected with a different MOI of live NiV (kindly obtained from Andrea Maisner) and incubated for 2 h. The inoculum was removed, and the cells were washed three times with PBS and left with 1 ml of DMEM/F12 supplemented with 2% FBS. At 24 h postinfection, the cells were washed once with PBS and incubated with 100% ethanol for 10 min at room temperature. After removal of the ethanol, nuclei were stained with Giemsa solution (1:10 dilution in water) at room temperature for 30 min. Excess staining solution was rinsed off with water, and the cells were allowed to dry for 10–15 min before evaluation of syncytia formation under a light microscope.

### Surface plasmon resonance.

A BIAcore 3000 instrument (Biacore, Piscataway, New Jersey, United States) was used to perform binding kinetics experiments. NiV-G-Fc diluted in sodium acetate (pH 4.0) was immobilized onto a carboxymethylated dextran (CM5) surface by standard amine coupling immobilization procedure. The ephrin analytes were diluted in HBS-EP buffers (BIAcore), and the injections were performed at a flow rate of 50 μl/min for 180 s. Surfaces were regenerated using 20 mM sodium hydroxide (BIAcore). Dissociation constants K_d_, K_on_ (association-rate), and K_off_ (dissociation-rate) were determined by fitting binding chromatogram data with BIAcore evaluation software (version 3.1) using the 1:1 Langmuir binding model.

## Abbreviations

CHO: Chinese hamster ovary
CNS: central nervous system
ELISA: enzyme-linked immunosorbent assay
Luc: luciferase
MOI: multiplicity of infection
NiV: Nipah virus
NiV-G: NiV attachment protein G
RFP: red fluorescent protein
VSV: vesicular stomatitis virus
